# Metabolic syndrome is associated with advanced prostate cancer in patients treated with radical retropubic prostatectomy: results from a multicentre prospective study

**DOI:** 10.1186/s12885-016-2442-7

**Published:** 2016-07-07

**Authors:** Cosimo De Nunzio, Giuseppe Simone, Aldo Brassetti, Riccardo Mastroianni, Devis Collura, Giovanni Muto, Michele Gallucci, Andrea Tubaro

**Affiliations:** Department of Urology, “Sant’Andrea” Hospital, “La Sapienza” University, Rome, Italy; Department of Urology, “Regina Elena” National Cancer Institute, Rome, Italy; Department of Urology, “San Giovanni Bosco” Hospital, Turin, Italy

**Keywords:** Prostate, Prostate cancer, Metabolic syndrome, High grade

## Abstract

**Background:**

Prostate cancer (PCa) is the most common non-skin cancer in USA and the second leading cause of cancer death in Western Countries. Despite the high mortality associated with PCa, the only established risk factors are age, race and family history. A possible association between metabolic syndrome (MetS) and PCa was firstly described in 2004 and several subsequent studies in biopsy cohorts have shown conflicting results. Aim of our multicentre prospective study was to investigate the association between MetS and PCa in men undergoing radical prostatectomy (RP).

**Methods:**

From January 2012 to June 2015, 349 consecutive men undergoing RP for PCa at three centres in Italy were enrolled into a prospective database. Body Mass Index (BMI) as well as waist circumference was measured before RP. Blood samples were also collected and tested for total PSA, fasting glucose, triglycerides and HDLs. Blood pressure was also recorded. We evaluated the association between MetS, defined according to Adult Treatment Panel III, PCa stage (advanced stage defined as pT ≥ 3 or N1) and grade (high grade defined as Gleason Score ≥ 4 + 3) using logistic regression analyses.

**Results:**

Median age and preoperative PSA levels were 66 years (IQR: 61-69) and 7 ng/ml (IQR: 5-10), respectively. Median BMI was 26.12 kg/m^2^ (IQR 24-29) with 56 (16 %) obese (BMI ≥ 30 kg/m^2^) patients and 87 (25 %) patients with MetS. At pathological evaluation, advanced PCa and high-grade disease were present in 126 (36 %) and 145 (41.5 %) patients, respectively. MetS was significantly associated with advanced PCa (45/87, 51 % vs 81/262, 31 %; *p* = 0.008) and high-grade disease (47/87, 54 % vs 98/262, 37 %; *p* = 0.001). On multivariable analysis, MetS was an independent predictor of pathological stage ≥ pT3a or N1 (OR: 2.227; CI: 1.273-3.893; *p* = 0.005) and Gleason score ≥ 4 + 3 (OR: 2.007, CI: 1.175-3.428; *p* = 0.011).

**Conclusions:**

We firstly demonstrated in a European radical retropubic prostatectomy cohort study that MetS is associated with an increased risk of high-grade and advanced prostate cancer. Further studies with long term follow-up should evaluate the impact of Mets on PCa survival.

## Background

Prostate cancer (PCa) represents the most common non-skin cancer in Western Countries and the second leading cause of cancer death. Notwithstanding the high mortality rate associated with PCa, the only established risk factors are age, race, and family history [1]. Large geographic disparity in PCa risk suggests that lifestyle factors may contribute to the aetiology of the disease. In fact, Asian men have incidence rates 10- to 15- fold lower than those observed in Western Countries, however PCa incidence in Eastern Countries and in emigrants has increased rapidly in the last years, suggesting that Westernization may represent an important etiologic factor [2].

Metabolic syndrome (MetS) was firstly described by Reaven in 1988 as complex disorder (*Syndrome X*), namely a constellation of metabolic abnormalities that increases the risk of coronary artery disease, cardiovascular atherosclerotic diseases and diabetes mellitus type 2 (DMT2) [3]. MetS has become a major public health problem in many Western Countries since its prevalence has been increasing; 35-41 % of adults in the USA are diagnosed with MetS [4]. According to the most widely accepted definition, proposed by the National Cholesterol Education Program’s Adult Treatment Panel III (ATPIII), patients with at least 3 of the following factors are considered to have MetS: abdominal obesity (waist circumference >102 cm in men or >88 cm in women), hypertriglyceridemia (>150 mg/dl), low high density lipoprotein (HDL) cholesterol (<40 mg/dl in men and <50 mg/dl in women), high blood pressure (>130/85 mm Hg) and a high fasting blood glucose level (>110 mg/dl) [1, 5].

Recently, increasing evidence supports the hypothesis that different metabolic factors and MetS may be involved in the development and progression of certain types of malignancies [6–8]. A possible association between MetS and PCa was firstly described in 2004 by Laukkanen et al [9] and several subsequent studies in biopsy cohorts have shown conflicting results [1, 10–18].

Aim of our multicentre prospective study was to evaluate the association between MetS, defined according to the ATPIII criteria, and PCa among a consecutive series of men undergoing radical prostatectomy (RP).

## Methods

From January 2012 to June 2015 a consecutive series of men undergoing RP for PCa at three centres in Italy were enrolled into a prospective database. The study was approved by the Ethics committee of the coordinator centre (Ospedale Sant’Andrea, La Sapienza University of Rome) and then of the Regina Elena National Cancer Institute, Rome, and San Giovanni Bosco Hospital, Turin. All patients signed a dedicated informed consent. Age and anthropometric parameters including waist circumference and body mass index (BMI) were assessed according to standardized methods and recorded from all patients. Waist circumference was measured, using a standard measurement strip with the patients standing and breathing normally, at the midway between the lowest rib margin and iliac crest [19]. BMI was calculated as weight in kilograms divided by height in meters, squared (kg/m^2^). Obesity was defined as BMI ≥30 kg/m^2^. Additionally, resting blood pressure was recorded as the first and fifth Korotkof sounds by auscultation methods [20]. Moreover, fasting (8 hours) blood samples were drawn from all patients during the preoperative assessment evaluation and analysed for blood glucose, HDL cholesterol, triglycerides, total Prostate Specific Antigen (PSA) [1]. Data were used to define a binary variable for the presence or absence of MetS, according to ATPIII [5]. Finally, prostate volume was evaluated before surgery by means of trans-rectal ultrasound.

As per European Association of Urology (EAU) Guidelines [21], surgical treatment was recommended to patients with a life expectancy of at least 10 years and a bioptic diagnosis of PCa, clinically localized or advanced (cT1-T3). Indication to surgery, independently from the presence of MetS, was proposed by a local muldisciplinary uro-oncology team evaluating all the prostate cancer cases diagnosed or referred to each hospital. One dedicated uro-pathologist in each centre performed the histological examinations of the RP specimens.

Pathologic report was standardized [22] according to the histological/architectural thresholds proposed by the World Health Organization (WHO) classification of tumor of the urinary system and male genital organs [23].

### Statistical analysis

Statistical analysis was performed using the Statistical Package for the Social Sciences (SPSS v.21, IBM Corp., Armonk, NY, USA). Evaluation of data distribution using the Kolmogorov–Smirnov test showed a non-normal distribution of the study data set. Differences between groups of patients in medians for quantitative variables and differences in distributions for categorical variables were tested with the Kruskal Wallis one-way analysis of variance and chi-square test, respectively. Using multiple logistic regression with the enter method, the statistical significant variables assessed in the univariate analysis were entered and investigated as predictors of advanced PCa (pathological Tumor stage ≥ pT3a and/or N1) versus localized PCA, and in a separate model predictors of high grade (Gleason score ≥ 4 + 3) versus low grade were compared. The logistic regression analysis was carried out using data from patients for whom complete data were available. In order to reduce the risk of redundant variables and subsequent multicollinearity, the variables included in the definition of the MetS were excluded from the multivariate analysis. An alpha value of 5 % was considered as threshold for significance. Data are presented as median (Inter quartile range (IQR)) and mean ± standard deviation (SD). Odds ratios (OR) and 95 % Confidence Intervals (CI) were calculated for the parameters in each model.

## Results

A total of 349 consecutive patients were enrolled (204 at Sant’Andrea Hospital, 78 at Regina Elena Institute and 67 at San Giovanni Bosco Hospital), with a median age and PSA of 66 (IQR: 61-69) years and 7 (IQR: 5-10) ng/ml respectively. Median BMI was 26.12 (IQR: 24-29) with 56 subjects (16 %) being obese. Baseline patients’ characteristics are summarized in Table 1. No significant differences (*p* > 0.05) for clinical and pathological characteristics were observed among the three centres (data not shown).

**Table 1:** Patient’s characteristics according to the presence or absence of Metabolic Syndrome

	Overall	No MetS	MetS	*p*
Patients (%)	349	262/349 (75 %)	87/349 (25 %)	
Age, years	64.5 ± 6.09 (66;61/69)	64.05 ± 6.53 (65;59/69)	64.92 ± 4.87 (65;62/68)	0.473
BMI, kg/m^2^	27.4 ± 12.77 (26.12;24/29)	26.56 ± 3.69 (26;24/28.4)	28.64 ± 3.45 (28;26.3/30.57)	<0.001
PSA, ng/ml	9.87 ± 11.84 (7.07;5.08/10)	10.41 ± 12.28 (7.11;5.1/9.8)	10.80 ± 16.75 (8.18;4.39/11.95)	0.687
TRUS Volume, ml	53.07 ± 29.59 (48;35.1/60)	55.43 ± 34 (47.75;36/60)	53.4 ± 24.01 (50;37/67)	0.661
Waist, cm	99.51 ± 9.85 (99;94/106)	98.87 ± 9.45 (98;93/105)	102.77 ± 11.3 (102.5;96/110)	0.009
Glycemia, mg/dl	101.86 ± 23.42 (96;88/108)	98.48 ± 19.15 (94;87/104)	113.19 ± 25.7 (111;97/111)	0.003
Triglyceridemia, mg/dl	131.8 ± 64.4 (115;83.7/161.2)	126.07 ± 58 (110;83/155)	152.11 ± 78.88 (133;87/199.5)	0.040
HDL, mmol/l	51.8 ± 14.06 (50;42/59)	52.87 ± 13.44 (51;43/60)	47.72 ± 15.9 (45;36.5/57)	0.023
Hypertension	182/349 (52 %)	113/262 (43 %)	69/87 (79 %)	<0.001

Data are presented as mean ± SD (median; IQR)

Metabolic Syndrome was diagnosed in 87 patients (25 %) according to ATPIII criteria. Patients with MetS showed higher BMI, waist circumference, fasting glycaemia, trygliceridemia and lower HDL. No statistically significant differences were observed between the MetS-group and the non-MetS-group regarding age (64.92 ± 4.87 years vs 64.05 ± 6.53 years; *p* = 0.473), serum PSA levels (10.80 ± 16.75 ng/ml vs 10.41 ± 12.28 ng/ml; *p* = 0.687) and prostate volume (53.4 ± 24.01 ml vs 55.43 ± 34 ml; *p* = 0.661). According to the National Comprehensive Cancer Network (NCCN) [24] and the preoperative available data (PSA, bioptic Gleason Score and clinical Stage), patients with PCa were classified into three categories: Low, Intermediate and High risk. Overall 134 patients (38.6 %) were diagnosed with a low risk cancer, 117 (33.7 %) with an Intermediate risk and 96 (27.7 %) with a High risk. Metabolic syndrome was significantly more prevalent (*p* < 0.001) in the Intermediate (30/117; 25.6 %) and High Risk (36/96; 37.5 %) population when compared with Low risk (21/134; 15.7 %).

Overall 126 patients (36 %) were found to have a non-organ confined prostate cancer (pT **≥** 3a) on pathological RP report (Table 2). Nineteen (5.4 %) patients presented positive lymph nodes (N1) and all of them had a ≥ pT3 PCa. No significant differences were observed between subjects with advanced and localized prostate cancers in terms of waist circumference (100.34 ± 9.22 cm vs 99.3 ± 8.13 cm; *p* = 0.503), BMI (27.07 ± 3.79 kg/m^2^ vs 26.64 ± 3.59 kg/m^2^; *p* = 0.149), glycaemia (103.87 ± 21.1 mg/dl vs 100.83 ± 19.68 mg/dl; *p* = 0.159) and HDL (50.78 ± 12.34 mg/dl vs 52.78 ± 12.34 mg/dl; *p* = 0.925). Patients with advanced prostate cancer were older (65.97 ± 5.97 years vs 63.44 ± 6.28 years; *p* = < 0.001) with a higher PSA (14.54 ± 17.84 ng/ml vs 7.81 ± 4.55 ng/ml, *p* < 0.001) and smaller glands (51.53 ± 29.86 ml vs 76 ± 44.05 ml, *p* < 0.001).

**Table 2:** Patient’s Characteristics according to pathological Stage and Gleason score

	< pT3a	≥ pT3a	*p*	GS < 4 + 3	GS ≥ 4 + 3	*p*
Patients (%)	223 (64 %)	126 (36 %)		204 (58.5 %)	145 (41.5 %)	
Age, years	63.44 ± 6.28 (65;59/68)	65.97 ± 5.97 (66;62/70)	<0.001	63.43 ± 6.16 (64;59/68)	64.69 ± 6.28 (66;61/69)	0.158
TRUS Volume, ml	76 ± 44.05 (60;44/96)	51.53 ± 29.86 (47;33.6/60)	<0.001	56.31 ± 31.57 (50;38/62.5)	53.23 ± 29.54 (49.5;35.5/57.9)	0.018
Waist, cm	99.30 ± 8.13 (98.87;96/102.77)	100.34 ± 9.22 (98.87;96/102.77)	0.503	99.21 ± 8.88 (99;94/105)	98.63 ± 10.89 (97;92/103)	0.549
BMI, kg/m^2^	26.64 ± 3.59 (26.98;24/28.65)	27.07 ± 3.79 (26.4;24/30)	0.149	26.51 ± 3.54 (26.98;24/28.4)	27.10 ± 3.8 (26.52;24/29.2)	0.131
PSA, ng/ml	7.81 ± 4.55 (6.8;4.79/9.43)	14.54 ± 17.84 (8.28;6.23/14.09)	<0.001	8.79 ± 8.16 (6.6;4.96/9.42)	13.22 ± 18.22 (8.7;6.15/11.7)	<0.001
Trygliceridemia, mg/dl	130.65 ± 63.4 (105;83/163)	136.23 ± 65.86 (122;86/161.5)	0.648	130.92 ± 50.74 (126;94/152)	135.25 ± 60 (126;94/152)	0.552
Glycaemia, mg/dl	100.83 ± 19.68 (98;91/104)	103.87 ± 21.1 (98;92/111)	0.159	100.19 ± 20.85 (95;88/107)	103.84 ± 23.15 (96;88/108)	0.153
HDL, mg/dl	52.14 ± 11.73 (52;45/56)	50.78 ± 12.34 (52;44/55.25)	0.925	52.1 ± 11.14 (52;46/55)	51.03 ± 12.99 (52;43/56.75)	0.213
Hypertension	102/223 (47 %)	74/126 (59 %)	0.037	88/204 (43 %)	91/145 (63 %)	0.001

Kruskal Wallis test for continuous variables, chi square test for categorical variables. *GS* = Gleason score

High grade PCa (Gleason Score ≥4 + 3) was diagnosed in 145 patients (41.5 %). Patients with high grade PCa were older (64.69 ± 6.28 years vs 63.43 ± 6.16 years; *p* < 0.001), with a higher PSA (13.22 ± 18.22 ng/ml vs 8.79 ± 8.16 ng/ml; *p* < 0.001) and smaller glands (53.23 ± 29.54 ml vs 56.31 ± 31.57 ml; *p* < 0.001) (Table 2).

MetS was significantly associated with advanced PCa (45/87, 51 % vs 81/262, 31 %; *p* = 0.008), pathologic nodal involvement (9/87, 10 % vs 10/262, 4 %; *p* = 0.028) and high grade PCa (47/87, 54 % vs 98/262, 37 %; *p* = 0.001). On multivariable analysis serum PSA levels and MetS were independent predictors of pathologic tumor stage ≥ 3a and Gleason score ≥ 4 + 3 (Table 3).

**Table 3:** Univariable and multivariable analysis for predicting advanced pathological stage and high pathological grade PCa

	Advanced pathological stage (≥ pT3a or N1)				High pathological Gleason Score (≥4 + 3)			
	Crude OR	*p*	Multivariable	*p*	Crude OR	*p*	Multivariable	*p*
Age, years	OR: 1.051 (CI: 1.010-1.094)	0.014	OR: 1.044 (CI: 0.997-1.094)	0.006	OR: 1.021 (CI: 0.984-1.060)	0.268	OR: 1.001 (CI: 0.961-1.044)	0.949
TRUS Volume, ml	OR: 0.994 (CI: 0.987-1.002)	0.155	OR: 0.993 (CI: 0.983-1.003)	0.173	OR: 0.997 (CI: 0.989-1.004)	0.356	OR: 0.998 (CI: 0.989-1.006)	0.594
PSA, ng/ml	OR: 1.068 (CI: 1.033-1.134)	0.001	OR: 1.121 (CI: 1.060-1.185)	0.001	OR: 1.038 (CI: 1.012-1.065)	0.004	OR: 1.064 (CI: 1.024-1.106)	0.002
BMI, kg/m^2^	OR: 1.016 (CI: 0.945-1.092)	0.673			OR: 1.027 (CI: 0.957-1.102)	0.459		
MetS	OR: 2.439 (CI: 1.492-3.987)	0.004	OR: 2.697 (CI: 1.481-4.913)	0.001	OR: 1.886 (CI: 1.164-3.054)	0.010	OR: 1.880 (CI: 1.066-3.316)	0.029
Waist, cm	OR: 1.016 (CI: 0.990-1.043)	0.218			OR: 1.009 (CI: 0.984-1.034)	0.494		
Glycaemia, mg/dl	OR: 1.007 (CI: 0.996-1.018)	0.203			OR: 1.007 (CI: 0.997-1.018)	0.181		
Trygliceridemia, mg/dl	OR: 1.001 (CI: 0.997-1.005)	0.565			OR: 1.002 (CI: 0.998-1.005)	0.423		
HDL, mg/dl	OR: 1.001 (CI: 0.983-1.019)	0.947			OR: 0.992 (CI: 0.974-1.010)	0.374		
Hypertension	OR: 1.621 (CI: 0.987-2.662)	0.056			OR: 2.180 (CI: 1.330-3.575)	0.002		

*OR* = Odds Ratio

## Discussion

In our series older patients with higher PSA levels and lower prostate volumes presented an increased risk of advanced and high grade PCa. These data were similar to previous experiences from our centre and from other studies demonstrating a negative correlation between prostate volume and PCa extent and aggressiveness [1, 25–29]. Specifically, Freedland et al reported a significantly higher incidence of advanced disease in men with small prostates [25]; Kassouf et al described that more poorly differentiated tumors were found at RP in subjects with small prostate volumes than in those with larger glands [28]. The biological mechanism accountable for the inverse association between prostate volume and cancer aggressiveness remains to be explicated. Since more PSA diffuses into the circulation from cancerous cells than from benign prostatic tissues, it is theorized that men with higher PSA density may have an increased risk of harbouring high-grade/advanced-stage PCa [30].

In the present study we observed that MetS is a common condition (87/349, 25 %) among patients undergoing RP and is more prevalent than obesity (56/349, 16 %). In a previous experience, we reported that MetS was even more prevalent (44 %) in patients at risk of PCa for an elevated PSA or an abnormal digital rectal examination and we also showed that MetS was associated with an increased risk (OR: 3.8; 95 % CI 1.33-10.9) of Gleason score ≥ 7 in patients with PCa at biopsy [1]. Our results have been later confirmed by Morote et al [14] (OR 1.75; 95 % CI 1.26-2.41) and Kayaly et al [12] (OR 1.8; 95 % CI 0.87-3.74) on similar bioptic studies. Notwithstanding all these studies, the association between MetS and PCa is still controversial. Most of the studies conducted on the European population have demonstrated a positive association between MetS and PCa incidence, aggressiveness and outcomes [9, 11, 13]. Similar cohort studies performed on Americans revealed null [17] or inverse [16] associations. Making a comparison between our series and the USA one may be misleading because of heterogeneity in age, race and BMI distribution. Our cohort is entirely Caucasian with neither Africans nor Hispanics and, although in line with the average Italian population, it is less obese (16 % vs 34 %) and older (median age 66 vs 62 years) when compared to other experience from USA series [1, 6, 31]. In 2013, Xiang et al conducted a meta-analysis confirming a weak association between MetS and PCa risk, although men with MetS appeared more likely to have a grater risk of biochemical progression after RP and a higher cancer-specific mortality [18]. Bhindi and colleagues recently highlighted that no individual MetS component is independently associated with PCa outcomes, however a correlation between the number of MetS components and the odds of PCa diagnosis exists, with a biologic gradient present [10]. Finally, data from a recent study based on the REDUCE (Reduction by Dutasteride of Prostate Cancer Events) population confirmed that having two or three to four MetS features was associated with increased risk of high-grade PCa (OR 1.35 and 1.94 respectively) [15].

One of the most important limitations and criticisms of the available evidence on the relationship between PCa and MetS is the lack of confirmatory studies on RP as most of the studies were performed on prostate biopsy cohorts where a significant percentage of patients with a negative biopsy unfortunately harbour a PCa. In order to overcome these limitations, we designed the first prospective multicentre study conducted on a consecutive series of patients undergoing RP using a standardized definition of MetS as the one proposed by ATPIII [5]. We observed that MetS is significantly associated with advanced PCa: patients with MetS presented a 2-fold increased risk of advanced stage and high-grade cancer. Our results are also supported by the retrospective single centre study presented by Kheterpal E et al [32], although data are not comparable since the author defined the presence of MetS using the criteria proposed by the International Diabetes Federation.

The biological mechanisms explaining these findings remain unknown. MetS features are known to accompany a pro-inflammatory state (elevated levels of C-reactive protein, TNF-α, interleukin 8, 6, 1β), which in turn has been related to prostate cancer risk [33–36]. Moreover, men with MetS are commonly diagnosed with hyperinsulinemia that has been associated with increased risk of PCa death. Finally, elevated circulating levels of IGF-1, leptin and adiponectin, commonly encountered in MetS patients, have all been associated with prostate cancer risk. However, current knowledge probably represents a minimal part of the biological mechanisms behind these associations and forthcoming studies are awaited [6].

We must acknowledge some limitations of our study. This is a multicentre study of patients with prostate cancer undergoing radical retropubic prostatectomy with a limited population of Caucasian men with no Africans or Hispanics; so far, our results cannot be extended to all patients with prostate cancer. Furthermore, a comprehensive evaluation of the association between MetS and PCa should include data on the different therapeutic options available for the management of T1-T3 PCa patients. However, although a trial investigating the impact of MetS in patients treated with External Beam Radio Therapy is ongoing in one of our centres and data will be soon available, the current study did not evaluate other possible treatment options. Considering the conflicting results reported by different authors when investigating the association between MetS and PCa in non-European populations, confirmatory findings from multicentre studies based on larger cohorts of patients of different ethnicities, receiving different treatment, are warranted.

Another limitation derives from the lack of information in the current study regarding physical activity or diet, which are associated with MetS, risk of PCa and potentially cancer grade at diagnosis [6]. Finally the lack of long-term oncologic outcomes, including at least biochemical recurrence free and metastasis free survivals does not allow supporting MetS as an independent predictor of poor oncologic outcomes. However this study is ongoing and results will be available in the near future.

Notwithstanding all these limitations, our study is the first prospective study evaluating patients treated with RP using the ATPIII criteria to define the presence of MetS. Our results, if confirmed, could open new issues in the management of patients with PCa and MetS as well as new studies investigating the role of physical activity, diet and medical treatment for MetS on PCa development and progression.

## Conclusions

In our multi centre study, we firstly observed that MetS is associated with an increased risk of high-grade pathological Gleason score and advanced pathological stage in patients with PCa treated with RP. Even though the molecular pathways are yet to be understood, it is assumable that metabolic factors should be considered as possible drivers of PCa differentiation and progression.

## Abbreviations

ATPIII, National Cholesterol Education Program’s Adult Treatment Panel III; BMI, body mass index; CI, confidence interval; DMT2, diabetes mellitus type 2; EAU, European Association of Urology; HDL, high density lipoprotein; IQR, Inter quartile range; MetS, metabolic syndrome; OR, Odds ratio; PCa, prostate cancer; PSA, Prostate Specific Antigen; REDUCE, Reduction by Dutasteride of Prostate Cancer Events trial; RP, radical prostatectomy; SD, standard deviation; TNF, Tumor Necrosis Factor; WHO, World Health Organization
